# Supplementary material for: Differential effects of cisplatin on cybrid cells with varying mitochondrial DNA haplogroups
Source: PeerJ. 2020 Oct 1;8:e9908. doi: 10.7717/peerj.9908 (PMC7533064; doi:10.7717/peerj.9908)
Supplement: Supplemental Information 1 — The mtDNA can be categorized into haplogroups according to accumulated SNPs. The map demonstrates mtDNA haplogroups associated with particular ethnic backgrounds throughout the world with their respective year of formation. Image courtesy of https://www.mitomap.org/foswiki/bin/view/MITOMAP/MitomapFigures. [file peerj-08-9908-s001.pdf]

Human mtDNA Migrations

From http://www.mitomap.org

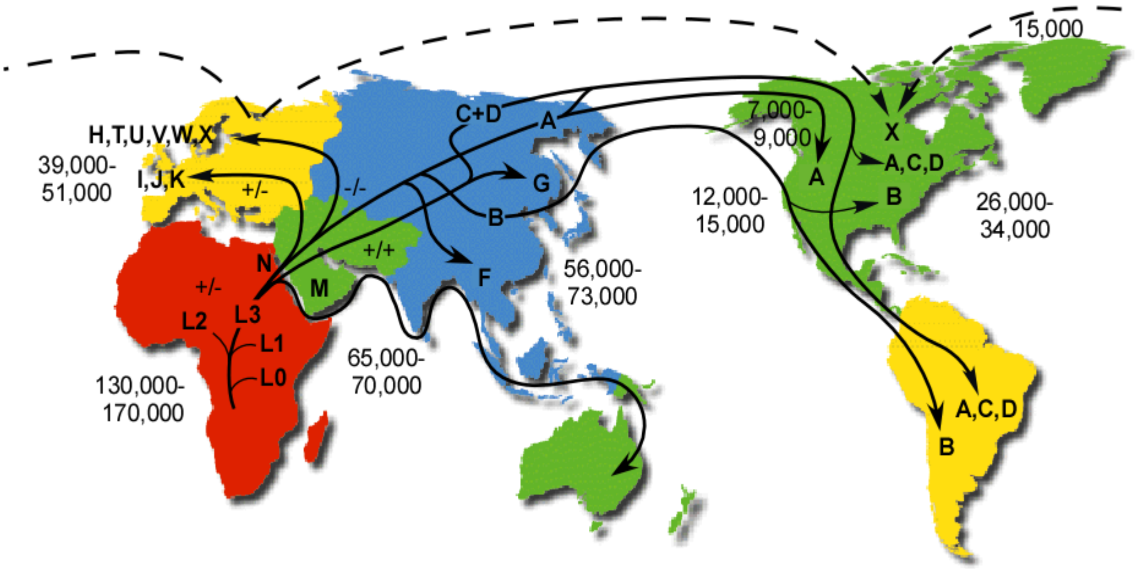

Symbols +/-, +/+, and -/- represent
RFLP status for Dde I 10394 / Alu I 10397

Mutation rate = 2.2 - 2.9 % / MYR
Time estimates are YBP

This is licensed by a Creative Commons Attribution 3.0 license.
